# Graph Neural Network Determine the Ground State Structures of Boron or Nitride Substitute C_60_ Fullerenes

**DOI:** 10.3390/nano15131012

**Published:** 2025-06-30

**Authors:** Linwei Sai, Beiran Du, Li Fu, Sultana Akter, Chunmei Tang, Jijun Zhao

**Affiliations:** 1 College of Mathematics, Hohai University, Changzhou 213200, China; 2 Guangdong Basic Research Center of Excellence for Structure and Fundamental Interactions of Matter, Guangdong Provincial Key Laboratory of Quantum Engineering and Quantum Materials, School of Physics, South China Normal University, Guangzhou 510006, China; 3 College of Mechanics and Engineering Sciences, Hohai University, Nanjing 210098, China

**Keywords:** heterofullerenes, graph neural network, Pólya’s enumeration theorem, deep learning

## Abstract

Substitutional doping of fullerenes represents a significant category of heterofullerenes. Due to the vast number of isomers, confirming the ground state structure poses considerable challenges. In this study, we generated isomers of C_60−*n*_B_*n*_ and C_60−*n*_N_*n*_ with *n* ranging from 2 to 12. To avoid overlooking the ground state structures, we applied specific filtering rules: no adjacent nitrogen (N) or boron (B) atoms are allowed, and substitutions in meta-positions within pentagons are prohibited when the substitution number *n* exceeds nine. Approximately 15,000 isomers across various values of *n* within the range of 2 to 12 for B and N substituted fullerenes were selected and optimized using density functional theory (DFT) calculations, forming our dataset. We developed a Graph Neural Network (GNN) that aggregates both topological connections and its dual graph with ring types as input information to predict their binding energies. The GNN achieved high accuracy, reaching a root mean square error (RMSE) of 1.713 meV. Furthermore, it operates efficiently; indeed, it can predict over six thousand isomers per second on an eight-core PC. Several predicted stable structures were further optimized by DFT to confirm their ground state configurations. The energy cutoffs of each composition were determined through statistical simulations to ensure that the selected ground state structures possess high confidence levels. Notably, new lower-energy structures have been discovered for boron-substituted fullerenes with substitution number ranging from seven to twelve and nitride-substituted fullerenes with substitution number ranging from seven to eleven.

## 1. Introduction

Heterofullerenes, which involve the incorporation of heteroatoms into the cage structures of fullerenes, can be classified into three categories: exo-doping, substitutional doping, and endo-doping. In comparison to pristine carbon fullerenes, these doped nanocages exhibit distinct electronic, magnetic, and optical properties, thereby expanding their potential applications in various fields such as catalysis [1], hydrogen storage materials [2], lithium battery cathodes [3], nonlinear optical materials [4,5], and molecular electronics [6,7,8]. Among all types of heterofullerenes, nitrogen- and boron-doped fullerenes have attracted significant attention due to the trivalent nature of nitrogen and boron atoms along with their comparable size and electronegativity to carbon. Notably, the chemical inertness of BCN materials surpasses that of diamond. Consequently, they are suitable for use in high-temperature semiconductor devices as well as short-wavelength optoelectronic devices [9,10].

Since the synthesis of carbon fullerene cages [11,12], there has been considerable interest in exploring their novel structures alongside their electronic and optical properties. In a pioneering experiment conducted in 1991 by Guo et al., boron-substituted fullerenes C_60−*n*_B_*n*_ (*n* = 1–6) were reported [13]. Chen et al. performed semiempirical and ab initio calculations on C_60_ substituted with two to eight B or N atoms [14] as well as on C_70_ substituted with two to ten B or N atoms [15]. They found that fullerene structures doped with fewer heteroatoms tend to exhibit greater stability.

In 2011, Garg et al. empirically identified several structures of C_60−*n*_B_*n*_ (where *n* = 1–12) and discovered potential lower-energy configurations [16]. They noted that boron-doped fullerenes contain no more than one B atom on a pentagon, while two B atoms can coexist in either para or meta positions on the same hexagon. Xie investigated a lower isomer of C_48_B_12_, which demonstrates enhanced third-order optical non-linearity, thereby suggesting its promising application in photonic devices [8]. Cheng conducted a systematic study of the C_60−*n*_B_*n*_ systems for *n* = 1–6 and uncovered new low-energy structures [17].

Comparatively, C_60−n_N_n_ azafullerenes have been studied more extensively than boron-doped fullerenes. Several azafullerenes, including C_59_N^+^ and C_69_N^+^ [18] and C_57_N_3_ [19], have been successfully synthesized in laboratory settings. Hultman et al. [20] discovered a cage structure of C_48_N_12_ exhibiting S_6_ symmetry and synthesized carbon-nitrogen nano-onions with a nitrogen content of 20%. A more stable configuration of C_48_N_12_ was theoretically proposed by Manna et al. [21], which retains the same S_6_ symmetry as reported by Hultman et al. [20]. Chen found that nitrogen substitution in host carbon fullerenes is generally more stable than boron substitution [14,15]. Sharma et al. [22] investigated the structural, electronic, and vibrational properties of C_60−*n*_N_*n*_ (*n* = 1–12). Srinivasu et al. [23] calculated the structure, stability, and nonlinear optical properties of C_60−2*n*_N_2*n*_ (for *n* = 1–12). In 2019, Cheng et al. [24] conducted a systematic investigation into the structures of C_60−*n*_N_*n*_ up to *n* = 12. They calculated all possible isomers for *n* = 1–4, estimated isomer energies for *n* = 5–9, and proposed a classification method to filter out unstable isomers for *n* = 10–12.

Despite the fact that some groups have investigated the substitution of B or N for carbon in C_60_ and other sized carbon fullerenes, the ground state structures of these heterofullerenes remain uncertain. This uncertainty primarily stems from the exponential increase in the number of isomers of C_60−n_X_n_ with respect to *n* [25], rendering it nearly impossible to calculate all possible isomers using first-principles methods. Generally speaking, previous studies have employed semiempirical approaches or applied strict filtering conditions to compute a limited set of isomers. However, excessive filtering may result in the exclusion of critical ground state structures [24].

In the past decade, machine learning (ML) techniques have been successfully employed to accelerate the structural prediction of clusters, including main-group clusters [26,27,28], coinage metal clusters [29], and specific cage clusters [30]. A ML model for atomistic simulations of boron and carbon, constructed using Gaussian approximation potential, can expedite the global minimum search for B_*n*_ (*n* = 36, 40, 84) and C_*n*_ (*n* up to 720) clusters. The global minimum structures of small clusters such as Pt_*n*_ (*n* = 8–14), Ta_*n*_ (*n* = 9–13), and Ag_*n*_ (*n* = 14–26) have been successfully obtained using various deep neural networks. Furthermore, global optimization efforts for larger Ag_*n*_ (*n* = 30–60) and Al_*n*_ clusters (*n* = 21–55) have demonstrated the scalability and transferability of machine learning methods.

Combining optimization techniques with machine learning could significantly enhance the current state-of-the-art in structural search methodologies. However, since these models rely on atomic coordinates as inputs, their accuracy is contingent upon a reasonable initial structure that typically necessitates DFT calculations. To further improve efficiency, it is desirable to develop effective methods capable of directly predicting energy based on topological connections within cage structures. A notable attempt in this direction was made by Liu et al., who utilized a neural network potential based on SchNet [31] to investigate the exohedral functionalization of fullerenes [30], achieving a mean absolute error (MAE) of just 0.37 eV. Moreover, in our previous work, we developed a graph neural network (GNN) model that directly predicts the binding energy of boron-nitride fullerene cages solely from their topological connections [32].

In this paper, we generated all possible isomers of C_60−*n*_B_*n*_ and C_60−*n*_N_*n*_ (*n* = 2–12) utilizing a recursive algorithm combined with isomorphic judgment techniques. Subsequently, we developed a modified GNN model to predict their binding energies both rapidly and accurately. This model employs ring type as initial features and performs convolution on both the source graph and dual graph to aggregate vertex and ring characteristics effectively. As a result, we confirmed several previous findings while also identifying some lower-energy structures.

## 2. Data Generation

In this study, we began with the well-known C_60_ buckyball with *I*_h_ symmetry and subsequently replace a portion of the C atoms with B and N atoms. Taking azafullerenes as an example, we first substituted a specified number (*n*) of N atoms into C_60_. The B-substituted fullerenes can be readily obtained by replacing all N atoms in the azafullerenes with B atoms. Given that all 60 C atoms are equivalent, we will consistently replace the first C atom with an N atom. Following this substitution, we will generate combinations represented by C(59, *n* − 1). Due to the high symmetry inherent in the structure of C_60_, some substitutions may yield equivalent configurations.

The symmetry group of C_60_ has been determined to be a group of order 120, consisting of permutations of the 60 atoms. For each permutation, a hash value is computed by encoding the replacement position using sexagesimal notation. The minimal hash among all 120 equivalent replacements is utilized to uniquely identify each substitution. During the generation of each combination, we verified whether it already exists in the hash set. If it does not exist, a new substitution is identified and its minimal hash is added to the set. Additionally, the replacement position is recorded as a valid structure. Each generated structure retains the minimum replacement position among its equivalent replacement isomers.

The theoretical number of isomers can be determined by Pólya’s enumeration theorem based on the symmetry group of the C_60_ structure. We generated the atom permutation group of C_60_ and then deduced that the number of replacement of *k* B or N atoms corresponds to coefficient of *x^*k*^* in following polynomial:

(1) $$\frac{1}{120}\left[{\left(1+x\right)}^{60}+16{\left(1+x^{2}\right)}^{60}+24{\left(1+x^{10}\right)}^{6}+20{\left(1+x^{6}\right)}^{10}+24{\left(1+x^{5}\right)}^{12}+20{\left(1+x^{3}\right)}^{20}+15{\left(1+x^{2}\right)}^{28}{\left(1+x\right)}^{4}\right]$$

The number of isomers for C_60_ substituted by N (or B) atoms is summarized in Table 1. As noted in previous studies [14,16], structures containing adjacent N or B atoms are energetically unfavorable. Consequently, we restrict our analysis to configurations where N and B atoms are not adjacent. When introducing a new N atom, its position and neighboring positions are excluded from the possible positions already in the set. The subsequent N atom is then placed in one of the remaining available positions. However, as the number of N atoms increases, the number of isomers without adjacent N atoms grows excessively large. For instance, when there are 10 N atoms, the number of isomers exceeds 10 million (see Table 1). Garg et al. also observed that a pentagon cannot accommodate more than one boron atom [16]. Furthermore, Srinivasu et al. [23] and Cheng et al. [24] excluded isomers with meta-position substitutions in pentagons and introduced additional filtering criteria to further reduce the number of isomers. In this study, we applied a rule prohibiting meta-position substitutions on pentagons for *n* = 10–12, which results in a tenfold reduction in the number of isomers (see Table 1). This filtering significantly enhances computational efficiency, allowing GNN to predict all isomer energies within an acceptable time frame.

Some structures of C_60−*n*_B_*n*_ and C_60−*n*_N_*n*_ were selected, with the number *n* ranging from 3 to 12. All isomers of C_57_B_3_ and C_56_N_4_ were included, while a subset of isomers for *n* > 4 was randomly selected. These structures were optimized with spin unrestricted for both open and close shell clusters using DFT as implemented in the DMol^3^ package [33], forming our dataset. The double numerical basis set along with the Perdew–Burke–Ernzerhof (PBE) functional within the generalized gradient approximation (GGA) [34] was employed for self-consistent field calculations. The relaxed C_60_-I_h_ structure yielded two distinct types of C-C bond lengths: 1.401 Å and 1.460 Å, which align reasonably well with experimental values of 1.401 Å and 1.458 Å [35,36]. Our dataset encompasses six compositions of C-B and C-N cluster systems: C_57_B_3_, C_54_B_6_, C_56_N_4_, C_54_N_6_, C_52_N_8_, C_51_N_9_, C_49_N_11_, and C_48_N_12_, and two test datasets—C_49_N_11_ and C_48_N_12_—while the remaining are designated as training data (Table 2). In total, the training and testing datasets comprise 11,594 and 1916 structures, respectively.

## 3. Train and Test

**Figure 1:**
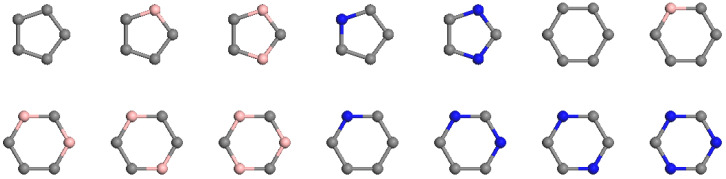
Ring types of all C-B and C-N pentagons and hexagons. Gray, pink and blue vertices are Carbon, Boron and Nitride atom respectively.

GNN is employed to predict the energies of C_60−*n*_B_*n*_ and C_60−*n*_N_*n*_ heterofullerenes. Based on our previous research, we found that utilizing the dual graph simplifies handling compared to its original graph [32]. Therefore, in this study, we adopted the dual graph as input. The first critical step is to the initial features for the vertices. We should emphasize that only topology information can be used as input because using coordinates would require optimizing structures first. However, optimizing millions of isomers using DFT calculations is impractical. Our task is to predict binding energies without relying on DFT calculations. In a simple graph, the degree or element type serves as a natural feature of a vertex; however, degree alone cannot differentiate between various isomers. Additionally, there is no element type present in the dual graph since each vertex corresponds to a ring in the original graph. In our prior investigation of (BN)_*n*_ fullerenes [32], we established that initial vertex features could be represented by permutations of neighboring vertices for each vertex, effectively capturing different rings. For C-B and C-N cage clusters, there are only 14 distinct types of pentagons and hexagons, as illustrated in Figure 1. Consequently, an integer ranging from 0 to 13 can be utilized to represent a vertex within the dual graph. This integer is then mapped to a vector through an embedding layer to form the feature vector corresponding to that specific vertex. Following this process, vertex features are updated by aggregating features from neighboring vertices into central vertices using various methods. Below are two approaches: one method involves summing all neighbor features [32,37] according to the following formula:

(2) $$H^{l+1}=\sigma (\hat{A}H^{l}W^{l})$$

Here, ***W*** represents a weight matrix used for transferring feature dimensions; ***H***^*l*^ denotes *l*-th layer of vertices’ features; σ is the active function; and *I* is identity matrix with the same order of ***A***. $\hat{A}$ signifies a matrix obtained from adjacent matrix ***A***.

(3) $$\begin{array}{l} \tilde{A}=A+I\\ {\tilde{D}}_{ii}=\sum_{j}{\tilde{A}}_{ij}\\ \hat{A}={\tilde{D}}^{-\frac{1}{2}}\tilde{A}{\tilde{D}}^{-\frac{1}{2}} \end{array}$$

An enhanced approach entails aggregating neighboring features with weighted coefficients, a technique commonly referred to as the attention mechanism [38]:

(4) $$\begin{array}{l} \alpha_{ij}=\mathrm{Softmax}(\mathrm{LeakyReLU}(h_{i}||h_{j}))\\ v_{i}^{l+1}=\mathrm{Elu}(h_{i}+\beta \sum_{j\in N(i)}\alpha_{ij}h_{j}) \end{array}$$

where ***h***_*i*_ represents feature of node *i*. α_ij_ is attention coefficients of node *i*. Softmax, LeakyReLU, and Elu are activation functions.

**Figure 2:**
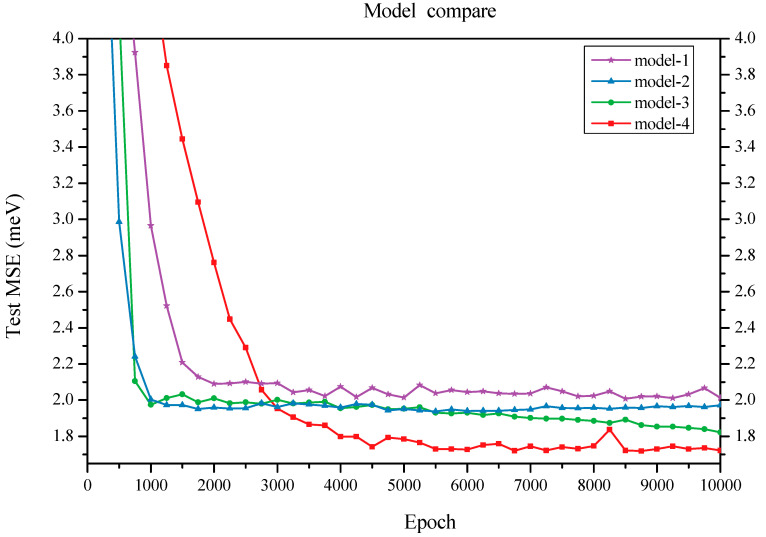
Comparison of test MSEs for four models.

We initially constructed a model that comprises a feature embedding layer, three graph convolution layers, a fully connected layer, and a final readout function. The feature embedding layer transforms the input ring type into vector features. The graph convolution layers capture local structural information. The fully connected layer maps each vertex feature to one dimension and subsequently sums all atom values into a single value that serves as the prediction for average binding energy. This model (referred to as Model-1) achieved a test mean square error (MSE) of 1.974 meV for average binding energy (see Table 3). Model-2 introduces an additional graph convolution layer following the three graph convolution layers from Model-1; this new layer aggregates dual-graph features onto the original graph vertices. As a result, this model attained a lower MSE of 1.932 meV (Figure 2). A more effective model (Model-3) was developed by incorporating element information: the elemental data from all 60 sites were embedded into vectors, followed by application of a *tanh* activation function and integration with another fully connected layer. Elemental information was added to vertex features prior to the readout function. Consequently, Model-3 achieved an even lower MSE of 1.822 meV.

When varying the number of graph convolution layers to two or four, we observed an increase in test MSEs to 1.862 and 1.872 meV, respectively; both results exceeded the test MSE obtained with three graph convolution layers. Furthermore, we compared the performance of graph attention layers against traditional graph convolution layers. By substituting two graph attention layers for the original ones in our architecture, we recorded a minimum test error of 1.713 meV for the resulting Model-4; however, employing three graph attention layers led to an increased test error of 1.868 meV (as shown in Figure 2), suggesting that two attention layers are sufficient. Overall, these findings indicate that networks utilizing ring type as input topology information alongside graph convolutional mechanisms for substructure feature extraction demonstrate robustness and exhibit minimal sensitivity to specific architectural details.

## 4. Prediction

Our GNN model has successfully and rapidly predicted all isomers of C_60−*n*_B_*n*_ and C_60−*n*_N_*n*_ for *n* = 4–12. The prediction speed reaches 6000 isomers per second on a PC equipped with an 8-core Xeon Gold 6139 CPU, allowing for the prediction of up to 2 million isomers within just 5 min. For any given system, the subsequent step involves selecting stable structures from the top list generated by the GNN predictions for further examination using DFT calculations, aimed at identifying the true ground state structure. Taking C_52_B_8_ as an example, we identified a total of 4,158,712 isomers, with the highest predicted binding energy being 8.5348 eV according to our GNN model. It is essential to determine a cutoff value E_c_ such that those isomers exhibiting predicted binding energies greater than 8.5348–E_c_ may potentially include the true ground state structure. Hence, only structures with predicted energies exceeding 8.5348–E_c_ will be considered for further analysis via DFT calculations.

It should be noted that both the distribution of predicted energies and the number of isomers can vary across different systems. To address this variability, we conducted 1000 simulations for each composition in order to ascertain its corresponding E_c_ value. In each simulation, we introduced random errors into the predicted energies of all isomers based on a distribution derived from sample statistics representing their true binding energy. Subsequently, we establish an E_c_ value such that in 5 out of 1000 simulations, the difference between the lowest predicted energy and simulated true energy remains less than E_c_.

**Figure 3:**
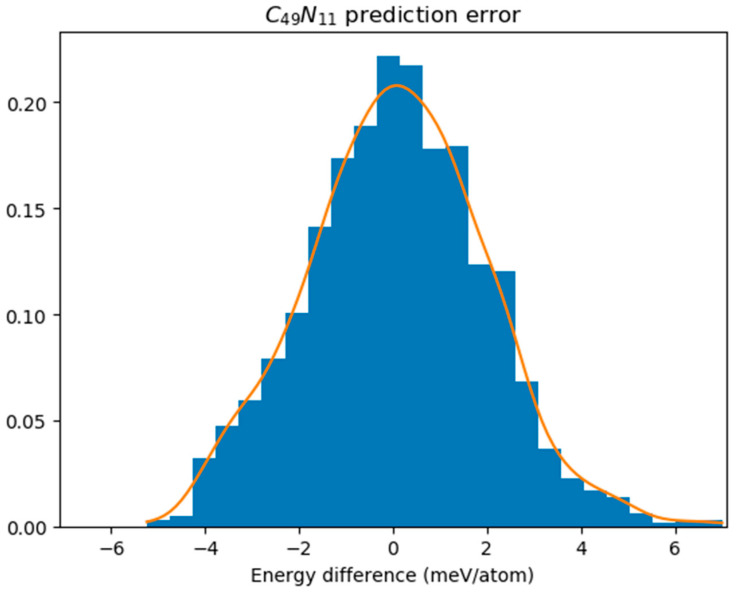
The distribution of energy difference between DFT calculation and GNN prediction for C_49_N_11_.

In Figure 3, we illustrated the energy differences between GNN predictions and DFT results specifically for C_49_N_11_ obtained from these simulations; this reveals a sample standard deviation (σ) of energy difference equal to 0.002147 eV. The prediction error was assessed using the Kolmogorov–Smirnov test, which yielded a *p*-value of 0.9802, significantly higher than our significance level set at 0.05. This indicates that we cannot reject the hypothesis asserting conformity to a normal distribution. We assume that discrepancies between GNN predictions and DFT results follow a normal distribution denoted as *N*(0, σ^2^). Accordingly, each GNN-predicted energy receives an associated random error ε~*N*(0, σ^2^). By selecting E_c_ = 3.5σ, only 91 isomers are predicted to have energies exceeding 8.5348–E_c_, leading to just four instances where the lowest DFT energy does not fall within this cutoff range. As a consequence, we can assert with 99.6% confidence that one of these 91 isomers corresponds to the ground state structure.

Table 4 presents all cutoff values and the number of isomers to be examined through DFT calculations for C_60−*n*_B_*n*_ and C_60−*n*_N_*n*_ with *n* = 4–12. In most cases, less than 1% of all possible isomers require evaluation. The selected combinations of cutoff values and isomers were optimized using DMol^3^ program with high precision. For *n* ≤ 6, our results align with previously reported ground state structures; however, for *n* > 7, new lower-energy structures were identified except in the case of C_48_N_12_.

**Figure 4:**
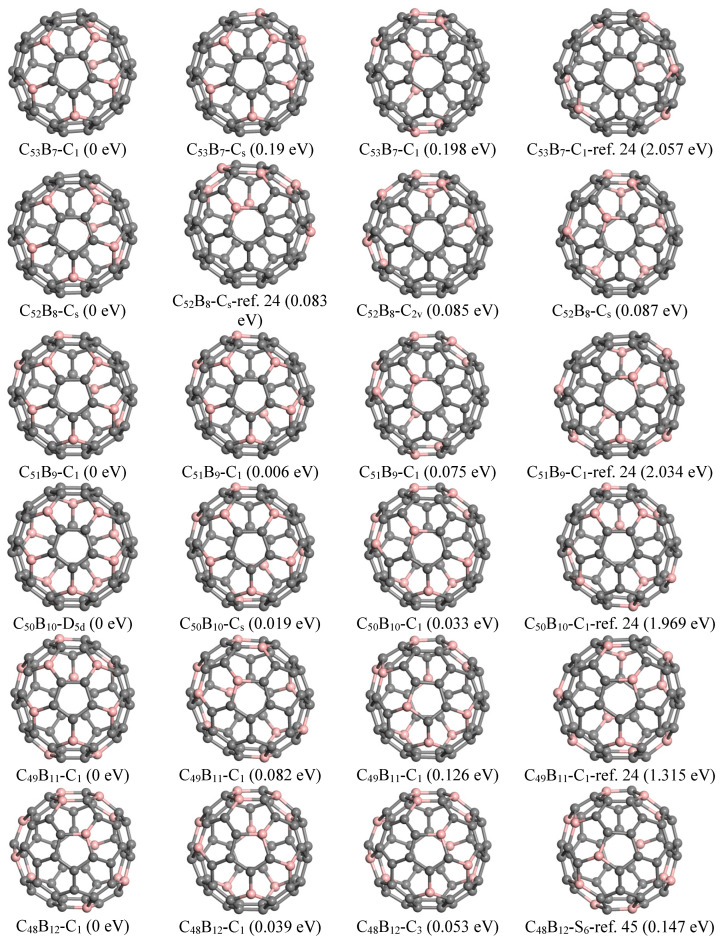
Predicted top three and reference structures of C_60−__*n*_B_*n*_ (*n* = 7–12).

The previously reported ground state structures and the top three lowest energy configurations identified by GNN for C_60−*n*_B_*n*_ and C_60−*n*_N_*n*_ with *n* = 7–12 are shown in Figure 4. The ground state structure of C_53_B_7_, as determined in this study, features C atom indices (1, 7, 11, 16, 24, 27, 36) that have been substituted with boron atoms. This configuration exhibits a flower-like substructure composed of five petal-shaped hexagons surrounding a pentagon; specifically, five B atoms are symmetrically arranged on the petals of these hexagons. Additionally, two other B atoms occupy para-positions within another hexagon, resulting in the formation of five pairs of para-positioned B-B bonds. Notably, this structure is energetically favored by 2.057 eV compared to that reported by Garg et al. [16]. The second lowest-energy configuration for C_53_B_7_ identified here has B positions at (1, 7, 11, 14, 24, 27, 31), which also maintains a similar arrangement involving five B positions.

Regarding the fullerene C_60−n_B_n_ series for *n* = 7, 9, 10, and 11, we have discovered several additional isomers exhibiting greater stability than those previously reported. The top five stable isomers are detailed in Table S1. For instance, the lowest-energy structure found for C_52_B_8_ corresponds to B positions (1, 7, 11, 15, 24, 27, 36, 39). It shares a comparable arrangement of five B atoms akin to that observed in C_53_B_7_ 7 while incorporating three additional boron atoms organized into two pairs located at para-positions within their respective hexagons. This particular configuration demonstrates an energy reduction of approximately 0.083 eV relative to isomer(b), which contains two B atoms situated within a pentagon as reported by Chen et al. [14], and it is also lower in energy by 1.014 eV compared to the structure presented by Garget al. [16].

The lowest-energy structure of C_51_B_9_ identified by GNN features B atoms located at positions (1, 7, 11, 14, 24, 27, 31, 36, and 39), exhibiting an energy reduction of 2.034 eV compared to the previously reported configuration [16]. This structure also includes a flower-like sub-structure akin to that found in C_53_B_7_; additionally, the remaining four B atoms are arranged in two para-positioned hexagons.

For C_50_B_10_, the ground state structure reveals a substitution pattern for B atoms at positions (1, 7, 11, 24, 27, 34, 37, and 50). It comprises two groups of five B atom substructures similar to those observed in C_53_B_7_ and demonstrates D_5d_ symmetry. Notably, this specific structure is energetically more favorable by approximately 1.969 eV when compared with earlier reported structure [16].

In the case of *n* = 11, the lowest-energy structure involves substituting B atoms at positions (1, 7, 11, 14, 17, 24, 27, 31, 35, 41, 57). This configuration showcases five B atoms positioned analogously to petal arrangements seen in C_53_B_7_. The remaining six B atoms are organized into three pairs situated in para positions. This particular geometry exhibits greater stability than the isomeric structure documented by Garg et al. [16], which contains a pair of meta-positioned B atoms within a pentagon and is less stable by approximately 1.315 eV.

For C_48_B_12_, we found that the most energetically favorable configuration consists of B atoms placed at locations (1, 6, 8, 11, 16, 18, 23, 28, 31, 36, 54, 60). In contrast to previous structures examined, it incorporates a hexagon containing three B atoms. Furthermore, the S_6_ symmetric structure reported by Manna et al. [39] is 0.147 eV higher in energy than our current ground state configuration and ranks fifth on our list.

**Figure 5:**
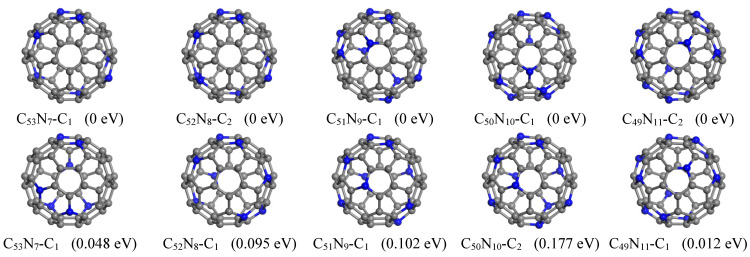
Lowest-energy structures of C_60−__*n*_N_*n*_ (n = 7–11) found by GNN (top panel) in comparison with the reported structures [24] (bottom panel). The relative energy is given after each structure.

In this study, we found lower energy structures for all C_60−*n*_N_*n*_ systems with *n* = 7–11, while the previously reported ground state structure for *n* = 12 has been confirmed (Figure 5). Our predicted lowest energy configuration of C_53_N_7_ features nitrogen atoms located at locations (1, 7, 26, 31, 37, 51, 54). This structure includes three pairs of para-positioned N and is energetically favored by 0.048 eV compared to the structure with two pairs of para-positioned N [24].

For C_52_N_8_, we identified eight structures (Table S1) that possess lower energies than those previously reported [24]. Among these configurations, the lowest-energy structure has N positioned at (1, 7, 26, 31, 37, 46, 51, 54), which is found to be energetically more favorable by 0.095 eV relative to that in literature [24]. The current ground state structure of C_51_N_9_ contains N atoms situated at positions (1, 7, 11, 14, 24, 27, 35, 54, 60). This configuration comprises four pairs of para-positioned N; this is in contrast to the previously reported one that contained only three pairs [24]. Our proposed structure is lower in energy by 0.102 eV in comparison to the previously reported structure featuring just three pairs of para-positioned N [24].

Furthermore, we have identified four additional structures with even lower energies. For C_50_N_10_, we discovered a total of sixteen structures that exhibit greater energetic favorability compared to those previously reported. Among these, the lowest-energy structure is favored by as much as 0.177 eV. This specific configuration comprises three pairs each of meta-positioned and para-positioned N atoms, which are substituted at positions (1, 6, 11, 15, 18, 43, 46, 49, 52, 56). In the case of C_49_N_11_, we found six isomers with lower energy. Notably, the ground state structure is 0.012 eV lower than the previously reported structure [24]. In this configuration, N atoms are positioned at (1, 6, 11, 18, 23, 27, 33, 40, 48, 51, 59), consisting of two pairs of meta-positioned N and six pairs of para-positioned N atoms.

## 5. Conclusions

To summarize, all possible cage structures of C_60−*n*_B_*n*_ and C_60−*n*_N_*n*_ with *n* = 2–12 have been generated. A graph neural network has been trained on over 10,000 data points, achieving a test mean squared error of 1.713 meV. This enables the rapid prediction of binding energies for a substantial number of isomers. Through meticulous statistical analysis, only the top several to thousands of isomers are selected to ascertain the true ground state structure for each system. New lower-energy structures have been identified for C_60−*n*_B_*n*_ with *n* = 7–12 and C_60−*n*_N_*n*_ with *n* = 7–11. Our methodology significantly accelerates the search for ground state structures in cage-like molecules or clusters and contributes to advancements in fullerene research.

## Figures and Tables

**Table 1:** Total and filtered number of C_60−*n*_N_*n*_ cages with *n* = 2–12.

n	Total Number	Adjacent Forbidden	Adjacent Forbidden and Meta-Postion Forbidden of Pentagon
2	23	21	
3	303	257	
4	4190	3019	
5	45,718	26,333	
6	418,470	180,316	
7	3,220,218	967,944	
8	21,330,558	4,158,712	
9	123,204,921	14,406,889	1,667,833
10	628,330,629	40,549,092	2,020,015
11	2,855,893,755		1,432,314
12	11,661,527,055		449,788

**Table 2:** Constitution of the dataset.

C_60−n_X_n_	C_57_B_3_	C_54_B_6_	C_56_N_4_	C_54_N_6_	C_51_N_9_	C_48_N_12_	C_52_N_8_	C_49_N_11_	Total
**Number**	257	1500	3019	2491	3175	1152	916	1000	13,510

**Table 3:** Model parameters and test error measured by mean square error (MSE).

Model	Feature Embedding Layer Size	Graph Aggregate Layer	Origin Graph Info.	Element Embedding	Test MSE (meV)
**1**	8	6,6,6	×	×	1.974
**2**	8	6,6,6	✓	×	1.932
**3**	6	6,6,6	✓	4	1.822
**4**	8	8,8	✓	4	1.713

**Table 4:** Energy cutoff and numbers of isomers for DFT calculations of C_60−n_B_n_ and C_60−n_N_n_ with *n* = 4–12.

Cluster System	Cutoff (σ = 2.147 meV)	Number of Isomers	Percentage of Total Isomers
C_56_B_4_	4 σ	19	0.629%
C_56_N_4_	3 σ	83	2.749%
C_55_B_5_	5 σ	995	3.779%
C_55_N_5_	2 σ	1217	4.622%
C_54_B_6_	4 σ	41	0.023%
C_54_N_6_	3 σ	1209	0.67%
C_53_B_7_	5 σ	8	0.0008%
C_53_N_7_	3.5 σ	991	0.102%
C_52_B_8_	3.5 σ	91	0.024%
C_52_N_8_	3.5 σ	6580	0.158%
C_51_B_9_	4 σ	76	0.005%
C_51_N_9_	4 σ	3588	0.215%
C_50_B_10_	σ	1	0.00005%
C_50_N_10_	5 σ	164	0.008%
C_49_B_11_	16 σ	294	0.021%
C_49_N_11_	3 σ	1364	0.095%
C_48_B_12_	8 σ	545	0.121%
C_48_N_12_	3 σ	6460	1.436%

## Data Availability

The code used in this paper is available in Gitee repository: https://gitee.com/saieuler/bcn_code. This open-sourced repository contains code generate structures, network definition, train file and other relevant codes. List of top 5 structures can be seen in Supplemental Information.

## Supplementary Materials

The following supporting information can be downloaded at: https://www.mdpi.com/article/10.3390/nano15131012/s1, Table S1: List and relative energy of isomers lower in energy than Reference. When isomer numbers larger than 5, only list top 5 isomers. Table S2: Cartesian coordinates (Å) of ground state C_53_B_7_, C_52_B_8_, C_51_B_9_, C_50_B_10_, C_49_B_11_ and C_48_B_12_ clusters. Table S3: Cartesian coordinates (Å) of ground state C_53_N_7_, C_52_N_8_, C_51_N_9_, C_50_N_10_, and C_49_N_11_ clusters.
