# Supplementary material for: Graph Neural Network Determine the Ground State Structures of Boron or Nitride Substitute C60 Fullerenes
Source: Nanomaterials (Basel). 2025 Jun 30;15(13):1012. doi: 10.3390/nano15131012 (PMC12251428; doi:10.3390/nano15131012)
Supplement: Supplementary file 1 [file nanomaterials-15-01012-s001.zip › nanomaterials-3657172-supplementary.pdf]

Supporting Information:

Graph Neural Network Determine the Ground State Structures of Boron or Nitride Substitute C_{60} Fullerenes

Linwei Sai [1,*], Beiran Du [1], Li Fu [2], Sultana Akter [1], Chunmei Tang [3] and Jijun Zhao [2,*]

[1] College of Mathematics, Hohai University, Changzhou 213200, China
[2] Guangdong Basic Research Center of Excellence for Structure and Fundamental Interactions of Matter, Guangdong Provincial Key Laboratory of Quantum Engineering and Quantum Materials, School of Physics, South China Normal University, Guangzhou 510006, China
[3] College of Mechanics and Engineering Sciences, Hohai University, Nanjing 210098, China

Table S1. List and relative energy of isomers lower in energy than Reference. When isomer numbers larger than 5, only list top 5 isomers.

Cluster size	B or N atoms positions	Relative energy compared with ref.	Ref no.
$C_{53}B_7$	00,06,10,15,23,26,35	-2.057	24
	00,06,10,13,23,26,30	-1.867	
	00,06,10,23,32,50,58	-1.859	
	00,02,06,10,16,23,26	-1.833	
	00,06,10,15,23,35,38	-1.823	
$C_{51}B_9$	00,06,10,13,23,26,30,35,38	-2.034	24
	00,06,10,13,23,26,30,37,52	-2.028	
	00,05,10,17,23,26,32,50,58	-1.99	
	00,06,10,13,42,45,48,51,54	-1.924	
	00,05,10,17,27,30,41,52,55	-1.919	
$C_{50}B_{10}$	00,06,10,23,26,33,36,49,53,59	-1.969	16
	00,06,10,13,23,26,30,37,52,55	-1.949	
	00,05,10,17,23,26,31,34,40,56	-1.936	
	00,05,17,29,32,42,46,50,54,58	-1.854	
	00,05,17,25,29,32,43,46,50,58	-1.822	
$C_{49}B_{11}$	00,06,10,13,16,23,26,30,34,40,56	-1.315	16
	00,06,10,13,16,23,26,30,34,53,59	-1.234	
	00,05,10,14,17,25,31,34,40,43,56	-1.19	
	00,05,07,10,15,18,22,35,39,47,57	-1.184	
	00,05,07,10,14,17,23,26,48,51,54	-1.182	
$C_{48}B_{12}$	00,05,07,10,15,17,22,27,30,35,53,59	-0.147	35
	00,05,07,10,14,17,22,26,31,34,40,56	-0.109	
	00,05,07,10,15,17,22,27,30,35,40,56	-0.094	
	00,05,10,13,17,25,30,43,46,51,54,58	-0.042	
$C_{52}N_8$	00,06,25,30,36,45,50,53	-0.095	25
	00,06,15,18,25,35,47,57	-0.052	
	00,06,10,14,23,34,53,59	-0.05	
	00,06,15,18,26,35,47,57	-0.047	
	00,06,10,23,30,36,50,53	-0.046	
$C_{51}N_9$	00,06,10,13,23,26,34,53,59	-0.102	25
	00,06,10,13,16,23,26,34,59	-0.042	
	00,05,10,17,23,26,35,49,59	-0.022	
	00,06,10,13,23,30,34,53,59	-0.024	
	00,06,10,23,26,33,36,49,53	-0.004	
$C_{50}N_{10}$	00,05,10,14,17,42,45,48,51,55	-0.177	25
	00,05,11,14,17,42,45,48,54,59	-0.159	
	00,05,11,14,17,22,45,48,51,56	-0.158	
	00,05,10,14,17,42,45,48,54,59	-0.135	
	00,05,11,14,17,42,45,48,51,55	-0.131	
$C_{49}N_{11}$	00,05,10,17,22,26,32,39,47,50,58	-0.012	25
	00,05,10,14,17,22,40,45,48,51,56	-0.011	
	00,05,10,14,17,23,26,40,48,51,56	-0.007	
	00,05,11,14,17,22,45,48,51,54,56	-0.005	
	00,05,10,17,22,26,30,32,39,50,58	-0.005	

Table S2. Cartesian coordinates (Å) of ground state $C_{53}B_7$, $C_{52}B_8$, $C_{51}B_9$, $C_{50}B_{10}$, $C_{49}B_{11}$ and $C_{48}B_{12}$ clusters.

		X	Y	Z
$C_{53}B_7$	C1	-0.457256124	3.837217485	1.403446096
	C2	0.726797146	3.412681080	0.705685879
	C3	1.945414999	3.801480397	1.492663703
	C4	1.550255580	4.495799032	2.690297588
	C5	2.242382620	4.279980801	3.896303621
	C6	-0.094623294	4.118600118	5.259010824
	C7	-0.807470451	4.351953263	4.039089591
	C8	-1.979662672	3.498435671	4.043826078
	C9	-1.600173201	3.011257939	1.402425917
	C10	-1.563765714	1.741274007	0.735297552
	C11	-0.419451841	1.370422388	-0.003924491
	C12	0.726138294	2.254016423	-0.077574891
	C13	3.210813894	1.786416449	0.696288458
	C14	3.106278636	2.997395746	1.490005395
	C15	3.802734141	2.761339907	2.742633766
	C16	3.349013962	3.356135334	3.925329608
	C17	3.330629050	2.571221837	5.163790999
	C18	2.229489430	2.968192998	6.009349467
	C19	1.529733736	1.998330843	6.747615160
	C20	-0.828221834	3.115137348	6.025855493
	C21	-1.994117243	2.745613755	5.281166114
	C22	-2.852982428	0.574324756	3.908788784
	C23	-2.858275320	1.310015819	2.710224719
	C24	-2.307456964	0.733543935	1.510651234
	C25	-1.855497229	-0.593855272	1.507187206

C26	-0.688172495	-0.974380487	0.746284019
C27	0.022283487	-0.028602040	-0.022290565
C28	1.461314628	-0.124337886	-0.140500975
C29	2.159862268	-1.086499385	0.597185483
C30	3.927988573	0.808687533	1.432551090
C31	4.262687278	1.385863170	2.720082671
C32	4.210651961	0.623075870	3.909104201
C33	3.761572604	1.232513633	5.136456820
C34	3.036156785	0.236359617	5.906458122
C35	1.916852631	0.608552120	6.662299862
C36	0.727787081	-0.261335870	6.674741576
C37	-0.478123118	0.525224368	6.773198963
C38	-1.613048572	0.163857433	6.033118456
C39	-1.572258798	-1.001232247	5.180331028
C40	-2.309397640	-0.761321605	3.930177638
C41	-1.859859924	-1.358005119	2.740707480
C42	-0.685035604	-2.197025850	2.733331689
C43	0.036107117	-1.974351296	1.502494570
C44	1.444018331	-1.990990472	1.471802151
C45	2.178826638	-2.211983525	2.725189815
C46	3.391150113	-1.428312975	2.734268419
C47	3.768070006	-0.782252685	3.920111362
C48	3.039173459	-0.988192194	5.143963772
C49	1.903149408	-1.820454935	5.150849036
C50	0.741194244	-1.450416254	5.926167803
C51	-0.433533894	-1.827186919	5.161299857
C52	0.018381563	-2.428591152	3.929193833
C53	1.461086757	-2.434516168	3.920473526
B1	-0.022147352	4.561550098	2.724372412

B2	1.444135398	4.097259498	5.242575064
B3	-2.428958654	2.821905620	2.730570538
B4	1.988716156	1.354518829	-0.150054375
B5	-0.042362380	2.040334894	6.810731385
B6	-2.437497864	1.273208765	5.261277376
B7	3.455542635	-0.653304444	1.383282027

		X	Y	Z
	C1	-0.458964053	3.826834973	1.423451713
	C2	0.719124972	3.415184541	0.701574494
	C3	1.934345699	3.797933023	1.479314224
	C4	1.547642160	4.485855043	2.690608459
	C5	2.251293745	4.259946714	3.885341940
	C6	-0.102372545	4.118257385	5.251453099
	C7	-0.813495455	4.350058780	4.043137087
	C8	-1.998460391	3.488906943	4.047018383
	C9	-1.609896455	3.001469496	1.421107587
	C10	-1.572013365	1.739900086	0.749528297
	C11	-0.424245326	1.372802752	0.001438396
	C12	0.715865514	2.263941787	-0.091765321
	C13	3.202150993	1.803662496	0.666540628
$C_{52}B_8$	C14	3.101046033	3.006558285	1.464141899
	C15	3.830652395	2.769000329	2.705451608
	C16	3.362256198	3.335718141	3.908389443
	C17	3.351830060	2.559342136	5.183639924
	C18	2.216273240	2.956138722	5.977134259
	C19	1.510884606	1.986750218	6.731524722
	C20	-0.837809350	3.108876672	6.018160957
	C21	-2.011313956	2.741701166	5.274247399
	C22	-2.886824350	0.566468041	3.906833806
	C23	-2.887975525	1.303506988	2.711394897
	C24	-2.325505981	0.729972659	1.515242749
	C25	-1.871870147	-0.595191481	1.509322205
	C26	-0.692346046	-0.963432520	0.757582834
	C27	0.018058490	-0.019141709	-0.017844566
	C28	1.457416363	-0.104057641	-0.125425800

C29	2.160806805	-1.048090574	0.631599505
C30	3.966064176	0.835367305	1.399446694
C31	4.365233453	1.426868086	2.643410176
C32	3.837991422	1.248198590	5.220730509
C33	3.045035122	0.245412529	5.903910842
C34	1.896303145	0.612486516	6.650464853
C35	0.703939136	-0.255400922	6.670089560
C36	-0.502257649	0.525670637	6.773129760
C37	-1.639128766	0.161359529	6.032673790
C38	-1.597583393	-1.002602634	5.181713961
C39	-2.333468972	-0.763379406	3.930243921
C40	-1.873887800	-1.357738078	2.739937851
C41	-0.696626449	-2.198135266	2.734875506
C42	0.028980364	-1.964821705	1.509486304
C43	1.437949585	-1.963734866	1.492652194
C44	2.167019270	-2.201691961	2.732199373
C45	3.410120991	-1.454284783	2.755541047
C46	3.854395740	-0.883067170	3.952711534
C47	3.056520287	-1.005597257	5.151164920
C48	1.889197378	-1.801848006	5.149791005
C49	0.720680455	-1.438377725	5.921237699
C50	-0.451490309	-1.818718623	5.159203856
C51	0.005375118	-2.424489765	3.928170633
C52	1.448040893	-2.425924352	3.923352531
B1	-0.022945612	4.558020490	2.732876306
B2	1.441038527	4.083998342	5.221408787
B3	-2.450889753	2.814932746	2.737972740
B4	1.979858601	1.374960248	-0.165515915
B5	-0.060947825	2.036403297	6.806035080

B6	-2.462605370	1.268382512	5.258000390
B7	3.477635658	-0.636414271	1.404383872
B8	4.390938247	0.591842513	3.935795394

		X	Y	Z
	C1	-0.493432182	3.833095156	1.429278990
	C2	0.735379630	3.388235210	0.749932049
	C3	1.896677719	3.784133795	1.524368918
	C4	1.525330872	4.488056759	2.727959763
	C5	2.248571702	4.269047719	3.910488812
	C6	-0.095467547	4.139187599	5.273986496
	C7	-0.821594189	4.385110771	4.062820675
	C8	-2.008089842	3.540501706	4.076941054
	C9	-1.663206575	3.061024645	1.424306645
	C10	-1.724389662	1.794526452	0.702659047
	C11	0.774321922	2.223620652	-0.043512191
	C12	1.924205649	1.353862387	0.013211934
	C13	3.085901064	1.743965937	0.726023735
$C_{51}B_9$	C14	3.043905804	2.970047587	1.499413299
	C15	3.801452735	2.746528939	2.704274040
	C16	3.344105383	3.331600012	3.917944142
	C17	3.355553107	2.563411868	5.191809330
	C18	2.239227976	2.969715435	6.010901528
	C19	1.539293791	2.004865984	6.750570618
	C20	-0.827204048	3.141310702	6.035974156
	C21	-2.010502486	2.780558805	5.295223502
	C22	-2.861044371	0.608811653	3.884604611
	C23	-2.924424902	1.364825484	2.712267077
	C24	-2.446063882	0.818438245	1.443976235
	C25	-0.658154924	-1.095153481	0.605387117
	C26	0.042925903	-0.130110318	-0.129031850
	C27	1.481209485	-0.070784585	-0.035496834
	C28	2.202891538	-1.048104087	0.664897775

C29	3.931723480	0.801285789	1.422939893
C30	4.357450375	1.408726383	2.634278437
C31	3.836474601	1.245216822	5.216285283
C32	3.063505428	0.245854758	5.907963213
C33	1.919163525	0.617826233	6.655049383
C34	0.730217801	-0.230522167	6.652905680
C35	-0.476981798	0.555192112	6.760159106
C36	-1.601011640	0.202912618	6.001135471
C37	-1.556432262	-0.958488257	5.141782679
C38	-2.287332491	-0.751048801	3.903598892
C39	-1.896736308	-1.414392947	2.725990370
C40	-0.677003680	-2.193438311	2.719470906
C41	0.060914206	-1.999035475	1.472662635
C42	1.475740999	-1.987384765	1.485746126
C43	2.211544364	-2.236585349	2.734741480
C44	3.458729068	-1.507735503	2.771873419
C45	3.892719079	-0.912598097	3.966229029
C46	3.084745195	-1.018717933	5.155966258
C47	1.922325320	-1.813224167	5.148918553
C48	0.747483078	-1.419808603	5.895828547
C49	-0.414961290	-1.790835008	5.130523201
C50	0.038380476	-2.412377236	3.910945323
C51	1.481518432	-2.434497367	3.920704084
B1	-0.046513276	4.571608976	2.748379027
B2	1.449087772	4.095668503	5.250398550
B3	-2.474574532	2.876404425	2.765762948
B4	-0.490613626	1.341779497	-0.129881686
B5	-0.038188181	2.060794104	6.812631005
B6	-2.437892982	1.303195718	5.241960935

B7	-1.956076639	-0.657634424	1.385348466
B8	3.504552192	-0.675861852	1.436802775
B9	4.397703643	0.573939292	3.928959340

		X	Y	Z
	C1	-0.482952055	3.871906629	1.404307596
	C2	0.719125233	3.439856070	0.737706847
	C3	1.907101956	3.825655455	1.510890263
	C4	1.528126133	4.525587137	2.710771061
	C5	2.231089054	4.299823269	3.904236228
	C6	-0.111151481	4.155190690	5.275093944
	C7	-0.833529278	4.392681577	4.049017574
	C8	-2.003012386	3.545472838	4.049200856
	C9	-1.616944903	3.046914748	1.404653075
	C10	-1.577427333	1.771295615	0.738250109
	C11	-0.445190798	1.377846071	-0.005228252
	C12	0.743417710	2.241045802	-0.004659089
	C13	1.945955464	1.452663139	-0.093042585
$C_{50}B_{10}$	C14	3.080461538	1.818912222	0.645376772
	C15	3.041165500	2.986944920	1.489597494
	C16	3.774973030	2.748099285	2.738326093
	C17	3.325803765	3.365349799	3.925595528
	C18	3.325390771	2.591300303	5.173510864
	C19	2.228367120	2.989400002	6.017961578
	C20	1.529896735	2.023643207	6.757653034
	C21	-0.831711756	3.161429126	6.033901605
	C22	-2.000039009	2.783570535	5.274408863
	C23	-2.858499398	0.598044537	3.903395394
	C24	-2.859119192	1.335922677	2.711358651
	C25	-2.311671484	0.760152887	1.509478128
	C26	-1.861488644	-0.576416359	1.490063468
	C27	-0.764465741	-0.974515617	0.645611049
	C28	-0.065995869	-0.008759025	-0.094080211

C29	2.295611319	-1.146541534	0.629674710
C30	3.463938233	-0.768684274	1.389167480
C31	4.322402728	1.416840030	2.760179144
C32	4.323022792	0.678961857	3.952216193
C33	3.775574198	1.254731605	5.154096729
C34	3.041329980	0.243588504	5.925323773
C35	1.909091844	0.637038610	6.668801339
C36	0.720484073	-0.226161145	6.668231983
C37	-0.482053613	0.562222429	6.756615513
C38	-1.616559872	0.195972873	6.018196528
C39	-1.577262874	-0.972061211	5.173977005
C40	-2.311070510	-0.733214641	3.925248146
C41	-1.861902170	-1.350465911	2.737978661
C42	-0.767187746	-2.284940433	2.759338997
C43	1.575052155	-2.140302513	1.388482447
C44	2.297428609	-2.377793525	2.614558021
C45	3.466911358	-1.530584970	2.614374101
C46	3.080846430	-1.032031027	5.258922024
C47	1.946854196	-1.857022735	5.259268050
C48	0.744776558	-1.424972684	5.925868241
C49	-0.443201361	-1.810772188	5.152685341
C50	-0.064225245	-2.510704456	3.952803880
B1	-0.048236153	4.598433522	2.735906783
B2	1.431709243	4.117250870	5.251931590
B3	-2.439679180	2.858876418	2.737036919
B4	-0.046012316	2.079921736	6.807655887
B5	-2.439238694	1.303278044	5.250237078
B6	1.509913400	-0.065036153	-0.144081433
B7	3.903140132	0.711608578	1.413338349

B8	0.032190596	-2.102364706	1.411643545
B9	3.903581011	-0.843990354	3.926538307
B10	1.512136200	-2.583548159	3.927668734

		X	Y	Z
	C1	-0.481843665	3.830730479	1.390555044
	C2	0.737303148	3.395406575	0.693986231
	C3	1.913646399	3.781117666	1.512103764
	C4	1.514851148	4.473084826	2.708571852
	C5	2.218623426	4.248266081	3.905795477
	C6	-0.106789438	4.138974652	5.275343394
	C7	-0.828296340	4.382835775	4.042876593
	C8	-1.996475752	3.556636223	4.055519698
	C9	-1.673296621	3.080086460	1.387750119
	C10	-1.754724963	1.826854360	0.668870242
	C11	0.741534378	2.263643857	-0.138269746
	C12	1.878730733	1.363512102	-0.188915521
	C13	3.086330141	3.000093866	1.479605419
$C_{49}B_{11}$	C14	3.747668297	2.707221320	2.722854743
	C15	3.305800899	3.310099053	3.930409767
	C16	3.343027326	2.552847986	5.173934206
	C17	2.222635521	2.958389946	6.032205906
	C18	1.510380644	2.033958693	6.817892368
	C19	-0.848969631	3.172487023	6.058213800
	C20	-1.998319136	2.790748966	5.301890373
	C21	-2.799570936	0.625355765	3.889332457
	C22	-2.891776737	1.382695975	2.708529941
	C23	-2.440214426	0.839543005	1.435187676
	C24	-0.650664960	-1.063217663	0.581825435
	C25	0.008847997	-0.106897650	-0.207919629
	C26	1.454595808	-0.008495914	-0.220067120
	C27	3.517370008	-0.656757741	1.468174707
	C28	3.941724342	0.675544281	1.470038127

C29	4.233175064	1.329172028	2.724047921
C30	4.221086676	0.611255917	3.944192728
C31	3.845856015	1.243016253	5.183812322
C32	1.883286068	0.628324007	6.846707095
C33	0.730791146	-0.210806853	6.849728164
C34	-0.497353906	0.577317622	6.833376857
C35	-1.579339841	0.201597696	6.036083400
C36	-1.530968552	-0.977000171	5.156474476
C37	-2.245518954	-0.729090056	3.917221827
C38	-1.850088443	-1.376021192	2.729850725
C39	-0.647746922	-2.156213121	2.715026250
C40	0.080307450	-1.992654406	1.464356802
C41	1.478539258	-2.053198791	1.465357947
C42	2.195650973	-2.213337865	2.712943033
C43	3.398493791	-1.376645712	2.719553284
C44	3.817441218	-0.799006905	3.938444368
C45	3.172933431	-1.137093959	5.185021648
C46	2.038200231	-1.954404516	5.181515087
C47	-0.440186808	-1.870362236	5.170243778
C48	0.057578274	-2.393860379	3.928523906
C49	1.517877250	-2.447910564	3.928471398
B1	-0.045881932	4.555818781	2.721308634
B2	1.437769494	4.076698543	5.257619169
B3	-2.463465772	2.895875477	2.745577838
B4	-0.537370060	1.374129535	-0.184769172
B5	3.156231871	1.721813943	0.606430725
B6	-0.070043248	2.100845298	6.851008999
B7	-2.394593666	1.302957337	5.253691409
B8	-1.926523812	-0.625745388	1.375347082

B9	2.275499274	-1.046466140	0.592092895
B10	3.141148268	0.171437208	6.051407513
B11	0.792128557	-1.538657362	6.050276565

		X	Y	Z
	C1	-0.532877773	3.910081889	1.355909411
	C2	0.682781643	3.455093346	0.714799578
	C3	1.860198295	3.913859896	1.480422432
	C4	1.475458284	4.621641457	2.648140520
	C5	1.538346607	4.152456757	5.301956025
	C6	-0.840415181	4.368169793	4.038069278
	C7	-1.909759153	3.449892042	4.030399998
	C8	-1.647093233	3.088559124	1.349025646
	C9	-1.610446947	1.790560982	0.707201503
	C10	-0.440006102	1.377851800	0.026517965
	C11	0.713478911	2.245597385	-0.025722728
	C12	3.272119902	1.768805184	0.603299641
	C13	3.965385952	2.802405007	2.770513251
$C_{48}B_{12}$	C14	3.515172629	3.386536692	3.958196123
	C15	3.372230936	2.608796569	5.158732344
	C16	2.210386182	3.089976414	5.944132341
	C17	-0.112719785	2.008426890	6.826943035
	C18	-0.812889143	2.986005968	6.114523805
	C19	-1.905919887	2.597583997	5.261983723
	C20	-2.359690382	1.268645233	5.215797280
	C21	-3.015510907	1.422986816	2.621927447
	C22	-2.425696895	0.839838854	1.470450157
	C23	-0.692299379	-1.035335714	0.688120016
	C24	0.011027108	-0.050773425	-0.016871733
	C25	1.438663295	-0.114142651	-0.145758215
	C26	2.145710415	-1.098655377	0.560029980
	C27	3.880849707	0.804880378	1.430923586
	C28	4.282796774	1.401404201	2.747836195

C29	4.239421970	0.634636289	3.928109277
C30	3.736941204	1.243743542	5.149840582
C31	3.017679778	0.229052747	5.955154076
C32	1.915131568	0.588884632	6.750130977
C33	0.724453882	-0.235968151	6.689550943
C34	-0.470891218	0.614026206	6.699330419
C35	-1.583382476	0.256567549	5.914327494
C36	-1.575732493	-0.997004803	5.159802984
C37	-2.361038647	-0.880964458	3.962534309
C38	-1.936807131	-1.497296608	2.772501593
C39	-0.710679504	-2.250910485	2.759858991
C40	0.044036126	-1.988841546	1.491701636
C41	1.455194959	-2.017075757	1.425576037
C42	2.273886249	-2.292874866	2.636513918
C43	3.489579807	-1.592319060	2.629401400
C44	3.081614697	-1.059290190	5.306503858
C45	1.953631440	-1.880413369	5.319901569
C46	0.734775872	-1.442998219	5.963511708
C47	-0.429682870	-1.821213812	5.163998360
C48	-0.021242442	-2.510629911	3.954181293
B1	-0.097240214	4.624765994	2.695336841
B2	2.287804335	4.396416987	3.942458900
B3	-0.017646920	4.126201977	5.355144270
B4	-2.475699229	2.869679812	2.683452192
B5	1.993165025	1.346509221	-0.148923384
B6	3.195965611	3.080001567	1.436142621
B7	1.449115928	2.053633436	6.814549669
B8	-2.930747440	0.599513979	3.919136487
B9	-1.968183791	-0.655273194	1.466688925

B10	3.458861824	-0.702470328	1.348119862
B11	3.870724126	-0.890153974	3.955126285
B12	1.514748101	-2.562534713	3.970077301

Table S3. Cartesian coordinates (Å) of ground state $C_{53}N_7$, $C_{52}N_8$, $C_{51}N_9$, $C_{50}N_{10}$, and $C_{49}N_{11}$ clusters.

		X	Y	Z
$C_{53}N_7$	C1	-0.418292284	4.442542954	1.514362606
	C2	0.737276457	3.784009985	0.749929426
	C3	1.888352788	3.437939145	1.515600736
	C4	1.433907285	3.802434492	2.735456404
	C5	2.130739916	4.378303544	3.928536130
	C6	0.059212489	4.149526853	5.094771262
	C7	-0.636793296	3.881715761	3.903466405
	C8	-1.801459435	4.102765262	3.913088542
	C9	-2.299536969	3.289142714	2.730353445
	C10	-1.578775214	2.734995960	1.504236023
	C11	-1.567458188	2.994448185	0.745527436
	C12	-0.432750260	1.764159184	-0.020648284
	C13	0.740636465	1.405393503	-0.012657732
	C14	1.904224803	2.237659150	-0.003050671
	C15	3.020151967	1.379389840	0.769994446
	C16	3.045025692	1.754476810	1.505131305
	C17	3.748687157	2.971238493	2.725809027
	C18	3.303712967	2.730791336	3.929283629
	C19	3.314251137	3.340572385	5.147478046
	C20	2.149506515	2.597276334	5.886675903
	C21	1.448770813	2.949306667	6.655856601
	C22	0.000255454	2.003853633	6.659660963
	C23	-0.658941491	1.980250646	5.852419016
	C24	-2.270724451	2.918160103	5.136306216
	C25	-2.739199583	1.238313098	3.939735106

C26	-2.739747430	0.647408554	2.719078154
C27	-2.269639778	1.364920152	1.516401605
C28	-0.666835354	-0.778669292	0.800370684
C29	-0.001279576	0.908725970	-0.000334164
C30	1.436393590	0.037764387	-0.000340901
C31	2.127728300	-0.011895913	0.805263524
C32	3.259755062	-0.908271075	1.564598144
C33	4.162742504	0.520201049	2.727254598
C34	4.157979568	1.368342656	3.921646481
C35	3.753897612	0.633148065	5.141730378
C36	3.041612297	1.243990490	5.906806353
C37	1.901587915	0.261560764	6.662826184
C38	0.739302725	-0.633221193	6.673628284
C39	-0.434620932	0.226712885	6.669480040
C40	-1.567560239	0.612387481	5.908611410
C41	-1.581733832	-0.986356115	5.165957046
C42	-2.296978353	-0.731414729	3.930930164
C43	-1.826309493	-1.291034120	2.735736467
C44	-0.676621213	-2.142983562	2.736580420
C45	0.034308254	-1.883568937	1.542032960
C46	2.122610671	-2.104507978	2.750139098
C47	3.251912291	-1.248280756	2.758832472
C48	3.019323002	-0.943481922	5.144117775
C49	1.906644807	-1.797245964	5.156586239
C50	0.745659391	-1.427802783	5.929873665
C51	-0.436538262	-1.817524942	5.174034805
C52	-0.003649520	-2.434588860	3.956719048
C53	1.430104993	-2.402046426	3.927404540
N1	0.004596969	4.442542954	2.696839756

N2	1.468336318	3.979749726	5.180217156
N3	-1.837713637	2.589407315	5.143017674
N4	-1.854342038	-0.575180820	1.508429447
N5	3.788258972	0.788836012	1.477447855
N6	1.453119613	1.930547591	1.505436758
N7	3.783954070	-0.745241522	3.962563897

		X	Y	Z
	C1	-0.425297914	4.431278668	2.704290189
	C2	0.730836206	3.777020048	1.521574820
	C3	1.882132811	3.434121776	0.756420764
	C4	1.429115679	3.804274303	1.523353105
	C5	2.125774240	4.380326730	2.740755970
	C6	0.056486311	4.149034861	3.936872414
	C7	-0.643066074	3.882348125	5.105264447
	C8	-1.813389569	4.092239217	3.907112707
	C9	-2.311926290	3.292103697	3.922027157
	C10	-1.585370530	2.730835777	2.730403334
	C11	-1.567238779	2.985800095	1.512427297
	C12	-0.428701980	1.755313408	-0.743702439
	C13	0.740601846	1.396221685	-0.012140846
$C_{52}N_8$	C14	1.902193185	2.236685615	0.006561512
	C15	3.017113566	1.378818446	0.003951343
	C16	3.035167473	1.755006260	0.771977052
	C17	3.740669120	2.968908043	1.511781459
	C18	3.297641988	2.728107435	2.729579546
	C19	3.309830989	3.337129361	3.933948377
	C20	2.149342150	2.592630988	5.151359013
	C21	1.446140972	2.949036364	5.891238603
	C22	-0.002403252	2.004137761	6.659568348
	C23	-0.659121394	1.983313966	6.669035512
	C24	-2.241463501	2.931437711	5.860825823
	C25	-2.705207092	1.241938490	5.132816499
	C26	-2.745828981	0.640454184	3.938044850
	C27	-2.260237678	1.360586852	2.713221669
	C28	-0.661778583	-0.769040358	1.520154323

C29	0.003476234	0.925010676	0.794014435
C30	1.434214763	0.030144791	-0.012854400
C31	2.132404155	-0.007593136	-0.004774048
C32	3.257243205	-0.909997341	0.803257373
C33	4.152292735	0.523298407	1.558556948
C34	4.155672640	1.363265621	2.726606436
C35	3.750091865	0.628362654	3.921940473
C36	3.040275897	1.237423212	5.141492930
C37	1.903305633	0.256736554	5.907181919
C38	0.741983455	-0.634898098	6.669679188
C39	-0.424740311	0.216471862	6.675324973
C40	-1.538443694	0.616398299	6.675780790
C41	-2.259501265	-0.705749889	5.871362584
C42	-1.821417954	-1.307386204	3.934566331
C43	-0.679671036	-2.145793942	2.730058532
C44	0.035731549	-1.894624673	2.726417037
C45	2.117971355	-2.118828553	1.530523667
C46	3.242119584	-1.254473001	2.741285233
C47	3.009977211	-0.948497012	2.755617930
C48	1.897286883	-1.805765727	5.140939664
C49	0.732799411	-1.410576181	5.149101004
C50	-0.421037534	-1.766046753	5.919907858
C51	-0.008157310	-2.408122960	5.145656918
C52	1.429264293	-2.419104833	3.951090328
N1	-0.002286913	4.431278668	2.704290189
N2	1.471180765	3.988481819	5.186690440
N3	-1.857641710	2.613410504	5.160804432
N4	-1.839496921	-0.574821075	1.523933978
N5	3.796270668	0.788861372	1.476864042

N6	-1.568871670	-0.949943874	5.140707591
N7	1.459992210	-1.952295051	1.501906424
N8	3.762736887	-0.748230247	3.958098624

		X	Y	Z
	C1	-0.409635426	4.461474555	2.708667166
	C2	0.719328082	3.786857306	1.553052816
	C3	1.894071603	3.409189036	0.785078308
	C4	1.427578329	3.792686976	1.531546085
	C5	2.127721075	4.382637162	2.741688933
	C6	0.045108816	4.159704194	3.940023096
	C7	-0.668459146	3.893541346	5.119415352
	C8	-1.805437369	4.127216986	3.912189842
	C9	-1.535163737	3.301054667	3.910709643
	C10	-1.515889496	2.938321638	1.554798962
	C11	0.728251666	1.739184175	0.815953573
	C12	1.886243507	2.210161178	0.036624095
	C13	3.068290179	1.382079087	0.039848562
$C_{51}N_9$	C14	3.065443340	1.778751019	0.733168435
	C15	3.776730308	2.994684066	1.503719392
	C16	3.296753245	2.755503121	2.729717385
	C17	3.293045545	3.339577388	3.927540322
	C18	2.126324687	2.587087048	5.148189549
	C19	1.423978136	2.958124951	5.876575423
	C20	-0.662242373	1.989870114	6.616984788
	C21	-1.799993225	2.930076823	5.868262658
	C22	-2.700963868	2.553384255	5.113430885
	C23	-2.695435733	0.652423668	3.938646013
	C24	-2.260690550	1.394050144	2.740382633
	C25	-1.888716176	-0.761023834	1.535502978
	C26	-0.720172761	-0.608430137	1.507009095
	C27	0.028226314	0.962374531	0.746987964
	C28	1.431044110	0.028700211	0.064688496

C29	3.270288943	-0.037125296	0.071189188
C30	3.760605173	0.544433549	1.539357303
C31	4.211528337	0.781295485	1.482424025
C32	4.166182086	1.372604237	2.725150981
C33	3.750070348	0.643100689	3.922763484
C34	3.027239566	1.240490071	5.150373197
C35	1.881955183	0.255354196	5.889828878
C36	0.726756925	-0.636296479	6.636602059
C37	-0.418583275	0.200973380	6.635391152
C38	-1.554049453	0.653638879	6.619536377
C39	-1.566115108	-0.942072929	5.874158094
C40	-2.284045896	-0.712948742	5.148173194
C41	-1.877416425	-1.352973939	3.930554737
C42	-0.691240774	-2.188808057	2.726728061
C43	0.014047559	-1.940851898	2.725219632
C44	1.424912241	-1.889217025	1.485148706
C45	2.115687274	-2.091369622	1.543437367
C46	3.248805665	-1.261440792	2.756142164
C47	3.011269398	-0.941566934	2.753977133
C48	1.880049994	-1.761635423	5.114525682
C49	0.735306432	-1.408469948	5.113065529
C50	-0.423773269	-1.790382541	5.889537362
C51	0.014237846	-2.373384793	5.149162311
N1	0.012760536	4.461474555	2.708667166
N2	1.458867115	3.989999456	5.175007092
N3	-2.338475503	2.755254117	2.703506894
N4	-0.418867998	1.385121581	0.007067276
N5	0.016210096	1.991219779	6.696810758
N6	-2.317729628	1.238181615	5.165338494

N7	2.161499224	-0.967775819	0.761669067
N8	3.806880250	-0.730701223	3.943352096
N9	1.436838053	-2.456941662	3.946985466

		X	Y	Z
	C1	-0.401228347	3.763004796	1.547077045
	C2	0.731036775	3.345328974	0.830082282
	C3	1.876779376	3.692818994	1.581148904
	C4	1.439316714	4.285439318	2.754327426
	C5	1.430595103	3.926381071	5.136721965
	C6	0.028049164	3.959262596	5.135363712
	C7	-0.688867635	4.150664862	3.913025904
	C8	-1.812940455	3.319035156	3.909335821
	C9	-1.563129750	2.952215855	1.547363521
	C10	-1.563464647	1.768610775	0.767941565
	C11	-0.417038668	1.382832854	0.035538795
	C12	1.881903123	1.397172654	0.041795329
	C13	3.012617425	1.762492363	0.791291768
$C_{50}N_{10}$	C14	3.718199322	2.742428861	2.730090754
	C15	3.275552450	3.331464032	3.909323203
	C16	3.302451171	2.585219773	5.125750318
	C17	2.151787455	2.971209871	5.890961812
	C18	1.440321624	1.985141413	6.644235502
	C19	0.007441349	1.976590998	6.625935535
	C20	-0.703889318	2.973581416	5.915680444
	C21	-1.850173148	2.590571926	5.158423840
	C22	-2.253998110	1.238008323	5.155323329
	C23	-2.692891025	0.628120975	3.930075119
	C24	-2.706211624	1.366094069	2.728648307
	C25	-2.288680322	0.768171297	1.520798005
	C26	-1.843000640	-0.579876044	1.537723956
	C27	-0.686139802	-0.952368382	0.775605761
	C28	0.015658027	0.030467106	0.011689192

C29	1.444756432	0.037643652	0.027554411
C30	2.140899644	-0.944566129	0.775723987
C31	3.305907410	-0.576569485	1.514158924
C32	3.740759916	0.783634054	1.513009482
C33	4.177693095	1.389207826	2.741988113
C34	4.208695362	0.640868662	3.939619505
C35	3.769339385	1.249356440	5.151481821
C36	3.019663139	0.247444067	5.904274245
C37	1.877068587	0.638837916	6.621324936
C38	-0.409979842	0.610747633	6.614547309
C39	-1.544106006	0.245990861	5.883992738
C40	-2.250913725	-0.726369079	3.938445473
C41	-1.823079113	-1.327883663	2.747482127
C42	0.022291039	-1.918721156	1.538717820
C43	1.426516353	-1.911949587	1.546584927
C44	3.294776529	-1.310018428	2.727404817
C45	3.738963639	-0.742956011	3.937594184
C46	3.023832307	-0.947353859	5.140248232
C47	0.714130832	-1.323940965	5.816688241
C48	-0.406206059	-1.671681893	5.090106988
C49	0.031203408	-2.278832697	3.885940480
C50	1.410986523	-2.294448193	3.899730089
N1	0.049079385	4.402045543	2.723712022
N2	2.176628225	4.272049915	3.964402143
N3	-2.294648685	2.733973530	2.719237095
N4	0.720432969	2.224236562	-0.019328865
N5	3.131531986	3.027255040	1.450631845
N6	0.752549610	-0.227812199	6.724324892
N7	-1.667965452	-1.016953980	5.214048716

N8	-0.745180983	-2.264867256	2.691689753
N9	2.188317799	-2.192919113	2.717176208
N10	1.853040702	-1.738983912	5.113448230

		X	Y	Z
	C1	-0.408682925	3.793153667	2.731175146
	C2	0.721525593	3.406008724	1.548125225
	C3	1.878622038	3.735416773	0.794682680
	C4	1.442858328	4.300202568	1.571877605
	C5	1.423621341	3.908185832	2.754699125
	C6	0.008113785	3.987500777	5.139030679
	C7	-0.678435343	4.144986097	5.138300680
	C8	-1.804849373	3.281767106	3.923257271
	C9	-1.545094458	2.957478680	3.907602034
	C10	-1.554504189	1.755933545	1.546564409
	C11	-0.416563202	1.389409524	0.783648908
	C12	0.711780272	2.219953672	0.028339076
	C13	1.880083355	1.377695457	0.011452771
$C_{49}N_{11}$	C14	3.024197416	1.745005175	0.037004776
	C15	3.723846774	2.735645964	0.772541203
	C16	3.260191588	3.305753520	2.709126277
	C17	3.232138760	2.532880280	3.914368500
	C18	2.107649551	2.919774442	5.086625491
	C19	0.017863050	1.991651720	5.852579172
	C20	-0.689826726	2.966093343	6.615361501
	C21	-1.815901919	2.550946485	5.896718597
	C22	-2.681332450	0.643370326	5.113471844
	C23	-2.724374197	1.400436996	3.927952221
	C24	-2.293626188	0.784226285	2.725538867
	C25	-1.797884805	-0.552434728	1.549310106
	C26	-0.646951782	-0.884892727	1.535488279
	C27	1.422723779	0.025244766	0.820302262
	C28	2.124345998	-0.947539839	0.044584970

C29	3.281769207	-0.574698447	0.776376339
C30	3.735759033	0.775173263	1.501474792
C31	4.173842945	1.394593868	1.504410093
C32	4.177701594	0.633847724	2.732897575
C33	3.008572505	0.281510442	3.926204033
C34	1.871792719	0.647500940	5.875873525
C35	0.733223075	-0.196631597	6.629844138
C36	-0.432042413	0.644707295	6.643039694
C37	-1.562272798	0.262610166	6.649115590
C38	-1.558943891	-0.950293550	5.881897229
C39	-2.238595220	-0.683522874	5.137538512
C40	-0.631691176	-2.074969885	3.916985929
C41	0.073711161	-1.831992548	2.754926371
C42	2.138089157	-2.150806791	1.586479960
C43	3.292075209	-1.328339290	2.724658624
C44	3.752727709	-0.718041785	2.725419061
C45	3.031712954	-0.942640892	3.931982457
C46	1.885067100	-1.783274639	5.147869718
C47	0.732602077	-1.417724034	5.134099301
C48	-0.407818876	-1.775685135	5.902290709
C49	1.430170099	-2.373016568	5.157117575
N1	0.038744655	4.436744943	2.731175146
N2	2.161865240	4.220213948	3.970755114
N3	-2.343712884	2.773890652	2.697342720
N4	3.096388506	2.999802851	1.468624058
N5	1.429058331	2.008225359	6.707067536
N6	-2.308815308	1.221863993	5.171318925
N7	-0.001456564	0.018161680	-0.076068157
N8	3.764506105	1.235387559	5.149237912

N9	-1.807437637	-1.315719819	2.733754827
N10	1.476622010	-2.020541531	1.466597418
N11	0.002291308	-2.443639730	3.974087843
